# Efficacy and safety of pregabalin and gabapentin in spinal stenosis: a systematic review and meta-analysis

**DOI:** 10.3389/fphar.2023.1249478

**Published:** 2023-11-29

**Authors:** Telmo Martínez, Gonzalo Mariscal, Jose Enrique de la Rubia Ortí, Carlos Barrios

**Affiliations:** ^1^ Institute for Research on Musculoskeletal Disorders, Valencia Catholic University, Valencia, Spain; ^2^ Department of Biomedical Sciences, Valencia Catholic University, Valencia, Spain

**Keywords:** lumbar spinal stenosis, pregabalin, gabapentin, gabapentinoids, treatment

## Abstract

**Background and Objective:** Multimodal management of spinal stenosis is on the rise, and central sensitisation inhibitors are playing an essential role in the treatment of central sensitisation processes. Pregabalin and gabapentin are antiepileptic drugs that decrease presynaptic excitability. The aim of this study was to investigate whether the use of pregabalin and gabapentin is effective in the symptomatic management of spinal stenosis, compared to other drugs, by using pain and disability rating scales. We also assessed the safety profile associated with these drugs.

**Methods:** We conducted a bibliographic search in the Pubmed, Web of Science, and Cochrane Collaboration Library databases. The inclusion criteria were studies that compared pregabalin or gabapentin to a control group in patients with lumbar spinal stenosis. We included randomized clinical trialsand a comparative retrospective cohort study. The primary clinical endpoints were VAS/NRS and ODI, measured at two, four, 8 weeks, and 3 months, while adverse events and walking distance were also collected. We combined the data using Review Manager 5.4 software.

**Results:** Our meta-analysis included six studies with a total of 392 patients, with a mean age of 60.3 years. We observed no significant differences in VAS scores at two, four, and 8 weeks: MD: 0.23, 95% CI: 0.63 to 1.09; MD: −0.04, 95% CI: −0.64 to −0.57; and MD: −0.6, 95% CI: −1.22 to 0.02, respectively. However, at 3 months, we found significant differences in favor of pregabalin with respect to VAS: MD: −2.97, 95% CI: −3.43 to −2.51. We did not observe significant differences respect to the ODI: MD: −3.47, 95% CI: −7.15 to −0.21. Adverse events were significantly higher in the pregabalin/gabapentin group (OR 5.88, 95% CI: 1.28–27.05).

**Conclusion:** Our meta-analysis suggests that abapentinoids may have a significant effect on VAS score at 3 months, but no significant differences were observed in ODI scores, and adverse events were higher in the gabapentinoids group.

## 1 Introduction

Lumbar Spinal Stenosis (LSS) is a degenerative condition that affects the spine’s structures, leading to compression of the thecal sac and nerve structures in the spinal cord and cauda equina. This condition impacts physical, psychological, and social aspects (Rampersaud et al., 2008; Ishimoto et al., 2013; Otani et al., 2013; Deer et al., 2019).

LSS is a common spinal disorder, with higher incidence in the elderly and those who are older or overweight. Symptomatic LSS is estimated to affect 11% of the general population, and up to 39% in clinical settings (Bagley et al., 2019; Kruger Jensen et al., 2020).

The most common symptoms of LSS include lumbar and radicular pain, neurogenic claudication, and incontinence. These clinical manifestations often require surgical interventions, making LSS the leading cause of spinal surgery in older adults (Deyo, 2010; Deyo et al., 2010; Genevay and Atlas, 2010). The degenerative process in LSS initially affects the intervertebral disc, leading to disc height reduction and herniation of the nucleus pulposusinto the spinal canal. This compresses spinal canal structures, generating stress on the lateral and posterior structures. Thickening and deformation of the yellow ligament result from fibrosis due to long-term mechanical stress. Surgical intervention is a therapeutic option to improve function in LSS patients (Weinstein et al., 2010; Shabat et al., 2011), however, treatment for LSS often begins with physiotherapy and pain control (Sengupta and Herkowitz, 2003; Lurie and Tomkins-Lane, 2016). Gabapentinoids (pregabalin and gabapentin) are a promising therapeutic option for LSS. These GABA analogue medications are commonly used to manage seizures but can also alleviate neuropathic pain by binding to voltage-dependent calcium and sodium channels. This mechanism reduces the release of excitatory neurotransmitters, such as substance P, glutamate, and noradrenaline, thus decreasing nerve excitability. This approach shows potential in managing LSS, which is often characterized by radicular pain and neurogenic claudication (Takahashi et al., 2014; Mathieson et al., 2020).

Gabapentinoids have been increasingly utilized for the treatment of pain, anxiety disorders, migraines, fibromyalgia, and restless leg syndrome, among other conditions. However, various adverse effects have been reported, particularly in the central nervous system. These effects include sedation, dizziness, gait instability, and feelings of toxicity. At therapeutic doses, dizziness or drowsiness has been reported in one of three patients who took these medications (Härmark et al., 2011; Derry et al., 2019).

This study aimed to investigate the effectiveness of pregabalin and gabapentin in managing spinal stenosis symptoms compared to other drugs, using pain and disability rating scales. It also aimed to evaluate the improvement in ambulation ability and safety profile associated with their use.

## 2 Material and methods

### 2.1 Information sources and eligibility criteria

**FIGURE 1:**
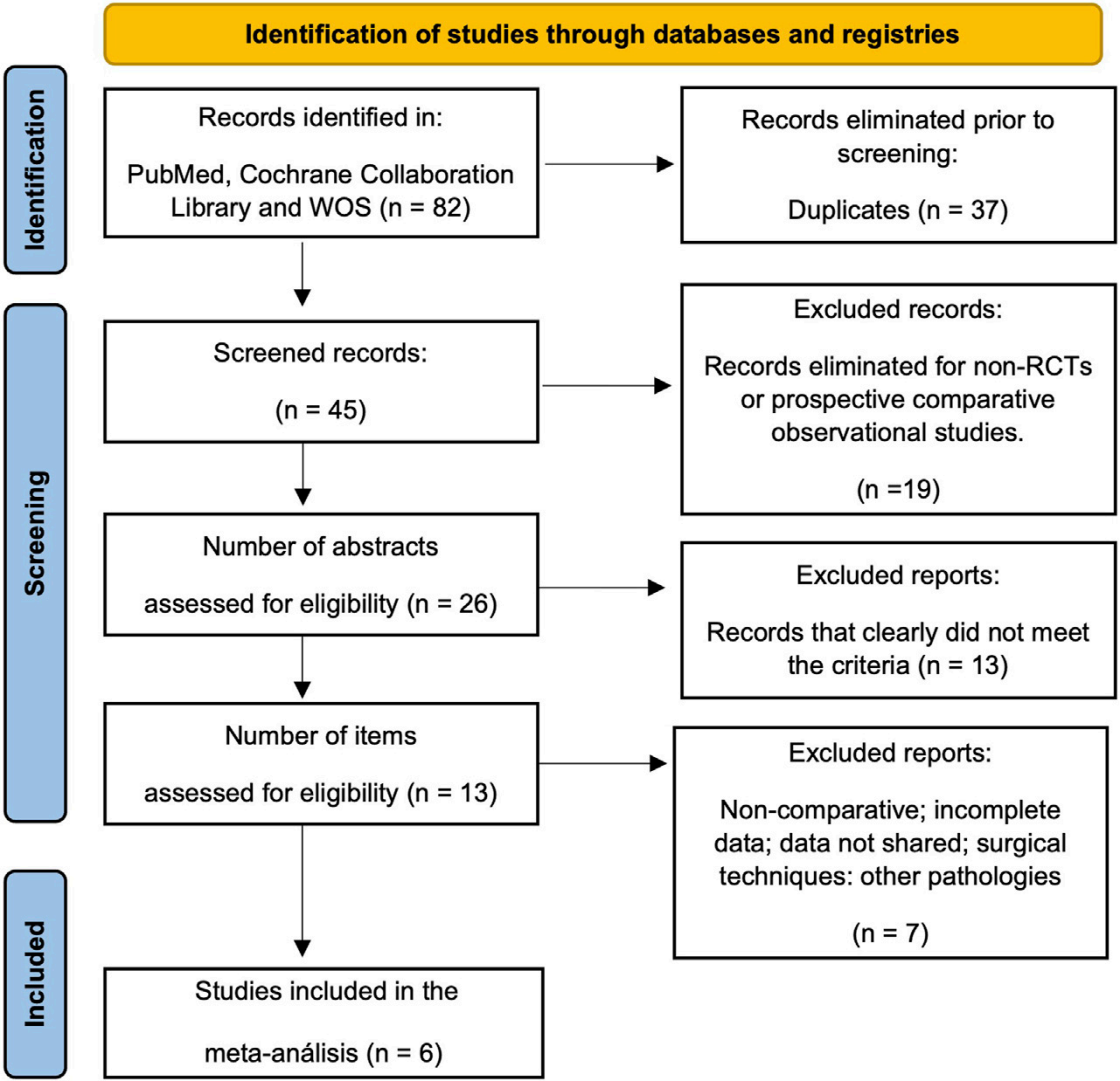
Study selection flow diagram (Preferred Reporting Items for Systematic reviews and Meta-Analysis).

This meta-analysis followed PRISMA guidelines (Preferred Reporting Itemsfor Systematic Reviews and Meta-Analyses) (Figure 1) (Liberati et al., 2009) and used the PICOS framework to establish inclusion and exclusion criteria. The population group included was adult patients with spinal stenosis, and the intervention was pharmacological treatment with pregabalin or gabapentin. The comparison was with alternative drugs, and the main outcomes were improvement in pain and disability, ambulation, and adverse effects. Comparative studies such as RCTs and cohorts were included, and exclusion criteria were duplicated or incomplete studies”.

### 2.2 Search methods for identification studies

The search strategy included the terms “Pregabalin” OR “Gabapentin” AND “Lumbar Spinal Stenosis,” without publication type screening. Randomized clinical trials and prospective observational comparative studies were included. The search was conducted in Pubmed, Cochrane Collaboration Library, and Web of Science. Two reviewers independently selected eligible studies and reached consensus on which to include. An initial screening of titles and abstracts eliminated obviously irrelevant studies, with full texts reviewed if needed. Disagreements were resolved through discussion.

### 2.3 Data extraction

Two authors independently reviewed the studies, and if consensus was not reached, a third author completed the data extraction form. General data extracted from RCTs included the number of patients, mean age, percentage of men, BMI, and follow-up period. Treatment regimens were also collected. The variables extracted for meta-analysis were divided into four groups: assessment of pain using Visual Analogue Scale (VAS), Numerical Rating Scale (NRS), disability assessment using the Oswestry Disability Index (ODI), and adverse events.

### 2.4 Assessment of risk of bias in included studies

**FIGURE 2:**
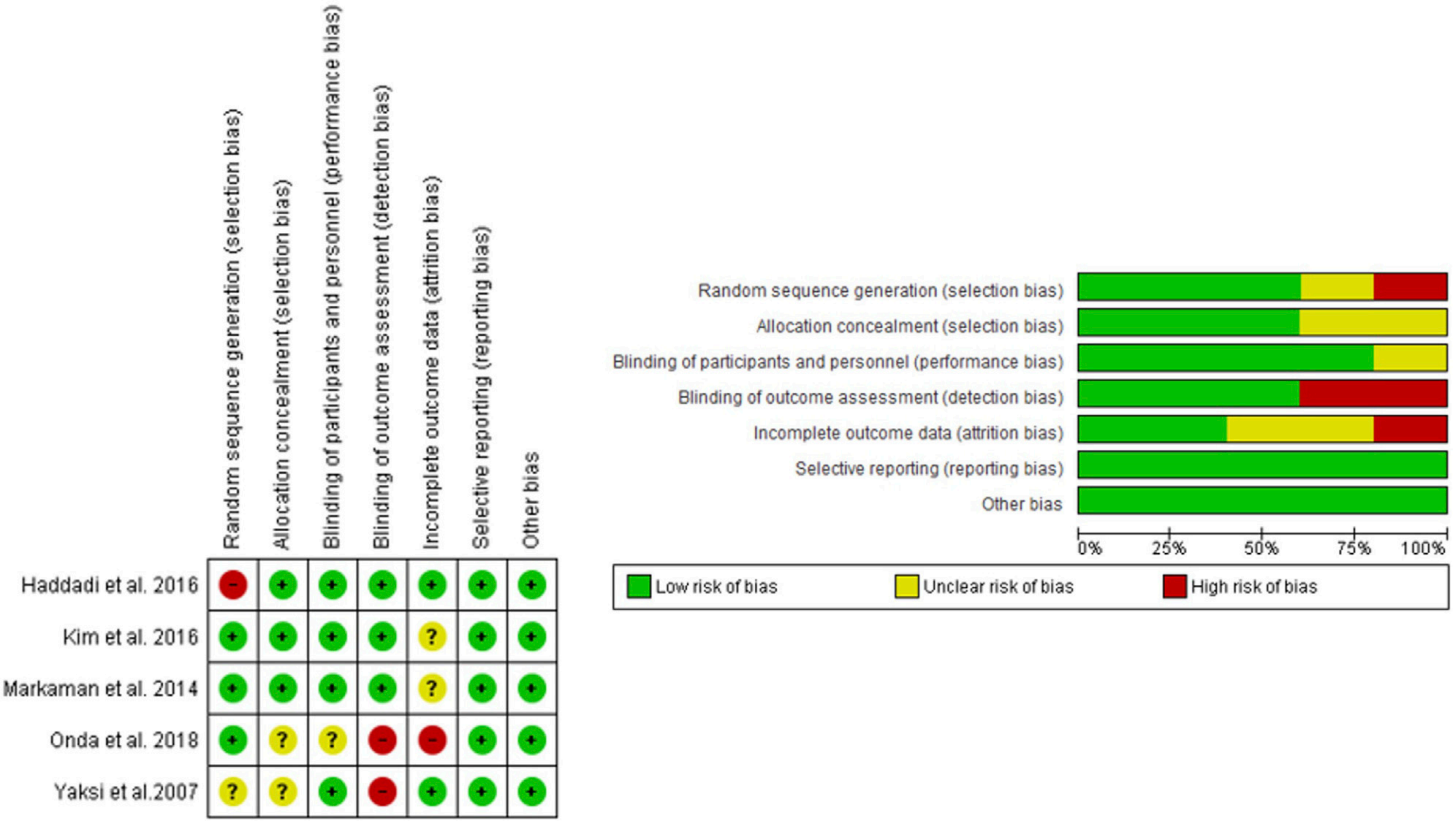
Risk of bias (green = low risk; red = high risk; yellow = unknown).

The quality of RCTs was evaluated by two reviewers using Review Manager, following six evaluation steps: random sequence generation, allocation concealment, blinding patients and personnel, blinding of data extraction, incomplete outcome data, and selective outcome reporting. The MINORS scale was used for one non-randomized controlled trial, with a maximum score of 24 for comparative studies (Figure 2). For non-comparative studies, scores of 0–4 were very low quality, 5–7 were low quality, 8–12 were fair quality, and ≥13 were high quality (Slim et al., 2003).

### 2.5 Statistical analysis

The meta-analysis was performed using the Review Manager 5.4 software package provided by the Cochrane Collaboration. Odds ratio (OR) with 95% confidence interval (CI) was calculated for dichotomous outcomes and mean difference (MD) with 95% CI was calculated for continuous variables. Heterogeneity was tested with both the Chi2 test and the I2 test. This ranges from 0% to 100%: values below 30%–40% represent no heterogeneity, between 30% and 60% indicate moderate heterogeneity, 50%–90% heterogeneity is substantial, and 75%–100% represents high heterogeneity. A fixed effects model was adopted if there was no statistical evidence of heterogeneity, and a random effects model was adopted if significant heterogeneity was observed. WebPlotDigitizer version 13.1.4 was used to extract accurate information from the figures in the articles.

Sensitivity analysis was performed to control for the influence of pregabalin and gabapentin separately, and was presented during the writing of the results.

## 3 Results

### 3.1 Study selection

A total of 82 records were identified in Pubmed, Cochrane Collaboration Library, and Web of Science databases. After eliminating 37 duplicate records, 45 records remained for screening. Nineteen were excluded as they were not RCTs or prospective observational comparative studies. The remaining 26 studies were screened by reading the abstracts, and 13 did not clearly meet the selection criteria. Reading the full texts of the remaining studies eliminated seven more that did not compare treatments, included surgical or non-pharmacological techniques, were incomplete, or did not address the pathology. (Figure 1) (Yaksi et al., 2007; Takahashi et al., 2014; Markman et al., 2015; Haddadi et al., 2016; Kim et al., 2016; Onda and Kimura, 2018).

### 3.2 Baseline data

The main characteristics of the six included studies are summarized in Table 1. A total of 392 patients were studied, with an average of 43.6% men in studies that provided this data. The overall mean age was 60.2 years, which was the same for both the control and experimental groups. Most studies that provided BMI data showed high values, with an overall mean of 30.04 kg/m2, and 30.55 kg/m2 in the control group and 29.53 kg/m2 in the experimental group. The total follow-up period varied across studies. Treatment regimens and administration times are compiled in Table 2. Figure 2 shows the risk of bias assessment of the RCT (Figure 2), and the MINORS criteria indicated fair quality for the non-randomized study.

**TABLE 1:** Main characteristics of the includes studies.

Study	n patients		Age		n man (%)		BMI		Follow up
	Control	Experimental	Control	Experimental	Control	Experimental	Control	Experimental	
Kim et al. (2016)	60	61	61.4 ± 9.4	62.9 ± 9.0	18 (30.0)	17 (29.5)	30.71 ± 4.3	25.02 ± 4.1	8 weeks
Onda and Kimura (2018)	17	18	61.0 ± 7.1	57.8 ± 12.0	12 (66.7)	12 (70.6)	23.7 ± 4.0	23.9 ± 2.8	8 weeks
Markman et al. (2015)	14	14	69 ± 8.7	71.1 ± 7.9	10 (67.0)	10 (71.0)	33.5 ± 5.4	30.7 ± 4.4	10 days
Takahashi et al. (2014)	47	49	68.5 ± 1.5	68.1 ± 1.6	22 (46.8)	27 (55.1)	_	_	3 months
Yaksi et al. (2007)	27	28	50.9 ± 10.5	50.7 ± 9.6	12 (44.0)	6 (21.0)	34.3 ± 8.6	38.5 ± 7.6	4 months
Haddadi et al. (2016)	30	27	51 ± 6.3	50.6 ± 6.8	_	_	_	_	8 weeks

**TABLE 2:** Treatment regimen applied to patients in each included study.

Study	Treatment schemes	
	Control	Experimental
Kim et al. (2016)	Limaprost (5 μg 3/d)	Pregabalin (75 mg 3/d)
Onda and Kimura (2018)	Limaprost (5 μg 3/d) + NSAID (standard)	Pregabalin (1^a^ sem. 25 mg 2/d, 2^a^ sem. 75 mg 2/d) + NSAID (standard)
Markman et al. (2015)	1. Dphenhydramine: 6.25 mg 2/d, 3 d - 12.5md 2/d, 7 d - 6.25 mg 2/d, 3 d Cleaning 7 d 2. Pregbalin: 75 mg 2/d. 3 días - 150 mg 2/d 7 d - 75 mg 2/d 3 d •	1. Pregbalin: 75 mg 2/d 3 días - 150 mg 2/d, 7d - 75 mg 2/d 3 d Cleaning 7 d 2. Diphenhydramine: 6.25 mg 2/d, 3 d - 12.5md 2/d, 7 d - 6.25 mg 2/d, 3 d
Takahashi et al. (2014)	NSAID (standard)	NSAID 2 s - NSAID + Pregabalin 25–50 mg/d 1 s - If not effective increase Pregabalin to 150 mg/d 1 s - If not effective increase Pregabalin to 300 mg/d.
Yaksi et al. (2007)	Conservative procedures + NSAID (standard)	Conservative procedures + NSAID + Gabapentin 900 mg/d. increasing 300 mg/week until 2400 mg/d
Haddadi et al. (2016)	Placebo	Gabapentin 300 md 3/d, 8 s Cleaning 4 s

### 3.3 Clinical results

#### 3.3.1 Visual analogic scale

**FIGURE 3:**
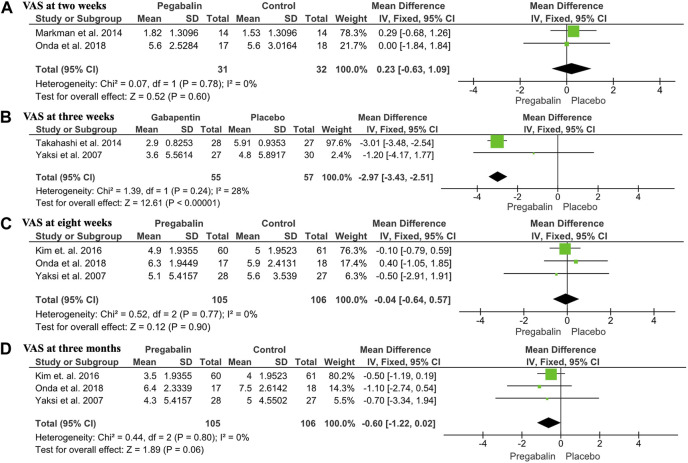
Forest plot showing VAS outcomes: **(A)** Forest plot showing the mean difference in VAS at 2 weeks between the pregabalin/gabapentin-treated group and the control group (MD: 0.23, 95% CI: −0.63 to 1.09, *p* = 0.6); **(B)** Forest plot showing the mean VAS at 4 weeks between the pregabalin/gabapentin-treated group and the control group in three studies (MD: −0.04, 95% CI: −0.64 to −0.57, *p* = 0.9); **(C)** Forest plot showing the mean VAS at 8 weeks between the pregabalin/gabapentin-treated group and the control group in three studies (MD: −0.6, 95% CI: −1.22 to 0.02, *p* = 0.6); **(D)** Forest plot showing the mean VAS at 3 months between the pregabalin/gabapentin-treated group and the control group in two studies (MD: −2.97, 95% CI: −3.43 to −2.51, *p* < 0.00001).

The VAS and NRS were used in five out of the six studies. Due to the chronology of data provided, it was difficult to interpret all the results, so they were subdivided into different temporalities. The mean difference with a 95% CI was calculated for this continuous variable. Markman et al. (2015) and Onda and Kimura (2018) provided data on VAS analysis for each patient at 2 weeks, with a total of 63 patients. The VAS obtained in the experimental and control groups was extracted and compared, showing no statistically significant difference between the two groups (MD: 0.23, CI 95%: −0.63 to 1.09, *p* = 0.6), with no heterogeneity (I2 = 0%) (Figure 3A).

At 4 weeks, three studies including 211 patients provided data on VAS means. Kim et al., Onda et al., and Yaksi et al. (Yaksi et al., 2007; Kim et al., 2016; Onda and Kimura, 2018) compared VAS means obtained from each patient in the group treated with pregabalin/gabapentin and the control group, with no statistically significant differences observed in any study (MD: −0.04, CI 95%: −0.64 to −0.57, *p* = 0.9), with no heterogeneity (I2 = 0%). The mean VAS obtained from the experimental group in the study by Yaksi et al. (Yaksi et al., 2007) was lower compared to the control group, while the one obtained in the study by Onda et al. (Onda and Kimura, 2018) was lower in the control group. In the study by Kim et al. (2016), the means of the VAS were practically the same (Figure 3B). When sensitivity analysis was performed, it was observed that gabapentin alone did not improve pain at 3 weeks (MD: −1.20, 95% CI: −4.17 to 1.77). In contrast, pregabalin improved VAS scores at 3 weeks (MD: −3.01, 95% CI: −3.48 to −2.54).

At 8 weeks, three studies including 211 patients reported data on VAS means. Kim et al., Onda et al., and Yaksi et al. (Yaksi et al., 2007; Kim et al., 2016; Onda and Kimura, 2018) compared the VAS means obtained from each patient in the group treated with pregabalin/gabapentin and the control group, reporting a lower mean VAS in the pregabalin/gabapentin groups. However, the results were not statistically significant in any of the studies (MD: −0.6, CI 95%: −1.22 to 0.02, *p* = 0.6), with no heterogeneity (I2 = 0%) (Figure 3C). The sensitivity analysis showed that neither gabapentin nor pregabalin analyzed separately improved pain at 8 weeks: gabapentin (MD: −0.50, 95% CI: −2.91 to 1.91) and pregabalin (MD: −0.01, 95% CI: −0.63 to 0.62).

At 3 months, two studies including 112 patients reported data on VAS. Yaksi et al. (2007) and Takahashi et al. (2014) compared the VAS meansobtained from each patient in the group treated with pregabalin/gabapentin and the control group, reporting a lower mean VAS in the pregabalin/gabapentin group, with statistically significant differences observed in both studies. The study by Takahashi et al. (2014) and overall result showed statistically significant differences in favor of pregabalin/gabapentin (MD: −2.97; CI 95%: −3.43 to −2.51; *p* < 0.00001), with heterogeneity (I2 = 28%) (Figure 3D). At 8 weeks, three studies including 211 patients reported data on VAS means. Yaksi et al. (2007); Kim et al. (2016); Onda and Kimura (2018) compared the VAS means obtained from each patient in the group treated with pregabalin/gabapentin and the control group, reporting a lower mean VAS in the pregabalin/gabapentin groups. However, the results were not statistically significant in any of the studies (MD: −0.6, CI 95%: −1.22 to 0.02, *p* = 0.6), with no heterogeneity (I2 = 0%) (Figure 3C). Sensitivity analysis showed that neither gabapentin nor pregabalin analyzed separately improved pain at 3 months: Gabapentin (MD: −0.70, 95% CI: −3.34 to 1.94) and pregabalin (MD: −0.59, 95% CI: −1.23 to 0.05).

#### 3.3.2 Oswestry disability index

**FIGURE 4:**
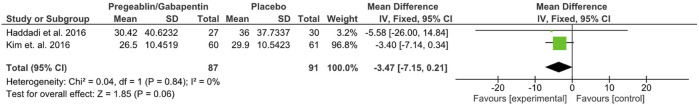
Forest plot showing the mean difference in ODI at 8 weeks between the pregabalin/gabapentin-treated group and the control group in two studies (MD: −3.47, 95% CI: −7.15 to 0.21, *p* = 0.06).

Three of the six included studies provided data on the ODI. Only two of these studies were comparable due to the chronology of data provided. The mean difference was calculated with a 95% interval for this continuous variable. Two studies including 178 participants reported data on the 8-week ODI. Haddadi et al. (2016); Kim et al. (2016) compared the ODI obtained from each patient in the group treated with pregabalin/gabapentin and the control group, reporting a lower mean ODI in the pregabalin/gabapentin group. However, the results were not statistically significant (MD: −3.47; CI 95%: −7.15 to 0.21; *p* = 0.06), with no heterogeneity (I2 = 0%) (Figure 4). The sensitivity analysis showed that there was no difference between using pregabalin or gabapentin separately: pregabalin (MD: −3.40, 95% CI: −7.14 to 0.34) and gabapentin (MD: −5.58, 95% CI: −26.00 to 14.84).

#### 3.3.3 Adverse events

**FIGURE 5:**
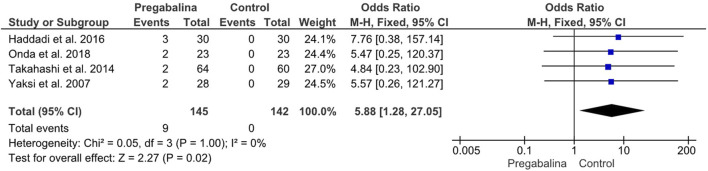
Forest plot showing the odds ratio of adverse events in the pregabalin/gabapentin-treated group compared to the control group in four studies.

All studies included in the meta-analysis reported adverse eventsresulting from treatment with each of the different drugs. Two studies were not evaluable due to providing the total number of adverse events instead of the total number of patients who suffered adverse events. Adverse events were analyzed as a dichotomous variable, and the Odds Ratio with a 95% CI was calculated. The four studies suitable for analyzing the total number of patients who suffered adverse events included 287 patients. Yaksi et al. (2007); Takahashi et al. (2014); Haddadi et al. (2016); Onda and Kimura (2018) provided the total number of patients who suffered adverse events, with a total of 9 patients reporting major adverse events, all of whom belonged to the group treated with pregabalin/gabapentin. A statistically significant difference in adverse effects was observed in the pregabalin/gabapentin treated group (OR 5.88, CI 95%: 1.28 to 27.05; *p* = 0.02), with no heterogeneity (I2 = 0%). Adverse events were classified as serious adverse events that could cause the patient to drop out of the study due to suffering from them (Figure 5). In the sensitivity analysis, there was no difference with respect to adverse events between pregabalin (MD:5.74, 95% CI:0.65–50.44) and gabapentin (MD:6.66, 95% CI:0.78–57.07).

## 4 Discusion

The research question addressed in this meta-analysis is important because gabapentin and pregabalin are widely used for the treatment of neuropathic pain by reducing the release of calcium to the nerve terminals (Takahashi et al., 2014). The symptoms produced by spinal stenosis have similar characteristics to neuropathic pain, and the available information about the efficacy of these drugs in treating spinal stenosis is mainly based on clinical practice. The literature on this topic is not extensive, and the effects can vary greatly from patient to patient. The aim of this meta-analysis was to compare various pain and disability scales and variables to assess the symptoms of lumbar spinal stenosis, to determine whether gabapentin and pregabalin are effective, and if they provide greater benefits than traditional drugs or placebos in treating the symptoms of spinal stenosis and slowing down its progression.

The visual analog scale or the numerical rating scale was one of the pain scores used in this study, and different results were obtained. After 2 weeks, the results from two studies were compared, and the mean difference with a 95% CI was calculated (−1.22 to 0.02). It was observed that after 2 weeks there was no significant differences. The MD obtained was 0.23, with no heterogeneity measured through an I2 of 0%. After 4 weeks, three additional studies were compared, including those by Yaksi et al. (2007); Kim et al. (2016); Onda and Kimura (2018). The studies by Yaksi et al. (2007); Kim et al. (2016) showed a slight improvement in pain with gabapentinoids, while the study by Onda et al. (Onda and Kimura, 2018) showed a slight improvement in the control group. However, when these results were compared, the difference was not statistically significant, with a 95% CI of (−0.64 to −0.57), an MD of −0.04, and no heterogeneity (I2 = 0%). At 8 weeks, none of the three studies showed a statistically significant difference between gabapentinoids and control groups. However, Onda et al. (Onda and Kimura, 2018) reported a slight improvement in the pregabalin group compared to the control group. After 3 months of treatment, a statistically significant improvement in pain was observed (MD: −2.97; 95% CI: −3.43 to −2.51; *p* < 0.00001), with heterogeneity (I2 = 28%) noted when comparing the studies by Yaksi et al. (2007); Takahashi et al. (2014). This improvement was reflected in a decrease in the VAS score. From the results obtained in this study, it appears that gabapentin/pregabalin did not improve pain as measured by the VAS/NRS when comparing the results at two, four, and 8 weeks. However, after 3 months, a statistically significant improvement was observed. One hypothesis could be that this was due to an insufficient treatment and follow-up period. This suggests that the beneficial effects may be produced in the long term than in the short term (Gonzalez-Escalada, 2005). Alternatively, different characteristics of the drugs used as control medications may have influenced the results.

Disability was assessed using the ODI, but only at 8 weeks, in the studies by Haddadi et al. (2016); Kim et al. (2016). The 95% CI was (−7.15 to 0.21), and the heterogeneity was measured through I2 of 0%. Although both studies showed that disability was slightly less in the experimental groups, the real difference was not significant when comparing both studies, with a mean difference of −3.47. However, studies that provided disability data through the Roland Morris Disability Questionnaire were not comparable. Two studies provided this data: Markman et al. (2015) showed that the control group had a lower disability index, although the difference was not significant. In contrast, Takahashi et al. (2014) reported significantly better results at 3 months with the use of pregabalin. This suggests that treating patients with gabapentinoids for a longer period may lead to better results and greater differences compared to those obtained with control treatments. After analyzing the results of the disability evolution obtained through these two indices, it cannot be concluded that gabapentinoids provide greater benefits than NSAIDs, Limaprost, or placebos.

Four of the six studies included in the meta-analysis provided information regarding claudication distance. However, due to differences in the way distance was expressed (either as an average or in intervals) and the variation in the timing of the results, it was not possible to make comparisons between the studies. The results of each study were analyzed separately. In the studies by Takahashi et al. (2014); Markman et al. (2015); Kim et al. (2016), the use of gabapentinoids did not lead to a greater walking distance until the patients were unable to continue. However, Yaksi et al. (2007) reported that the addition of pregabalin to the use of NSAIDs resulted in a greater walking distance in the experimental group until the patients were unable to continue, compared to the distance traveled at the beginning and halfway through the treatment, and with NSAIDs used alone.

The final variable analyzed in this study was the incidence of adverse events experienced by the patients during the follow-up period. Data on adverse events were provided by all six studies, but only four were comparable, as the other two studies reported the total number of adverse events instead of the number of patients who experienced them. Comparing the four studies by Yaksi et al. (2007); Takahashi et al. (2014); Haddadi et al. (2016); Onda and Kimura (2018), it was observed that the incidence of adverse events was significantly higher in patients treated with gabapentinoids when compared to the control group (OR: 5.88, 95% CI: 1.28 to 27.05, *p* = 0.02), with no heterogeneity (I2 = 0%). Most of the patients who reported experiencing adverse events had to withdraw from the study, indicating that the use of gabapentinoids may not be suitable for patients who cannot tolerate their potential adverse effects. In contrast, Kim et al. and Markman et al. did not provide comparable data on adverse events, while Haddadi et al. and Takahashi et al. reported significantly higher incidence of adverse events in patients treated with gabapentinoids (OR: 5.88, 95% CI: 1.28 to 27.05, *p* = 0.02). However, Markman et al. (2015); Kim et al. (2016) provided a more complete record of adverse events and how many participants experienced them. Both studies identified the most frequent symptoms as those affecting the central nervous system, with dizziness being the most common. Although the two studies were not comparable, both showed that the total number of adverse events was significantly higher in the experimental groups. Kim et al. reported a difference of 30-9, while Markman et al. reported a difference of 37-13. These results suggest that other treatments, which may seem less effective in advance, may provide a higher benefit to patients who cannot tolerate the adverse events associated with gabapentinoids.

The findings of this study underscore the importance of multimodal conservative treatment in patients with lumbar spinal stenosis. A combined approach using medications such as gabapentin or pregabalin, along with individualized physical therapy and therapeutic exercises, may offer optimal results for many patients before surgical decompression is considered. Future research should aim to provide more detailed specifications of the conservative treatment protocols applied, in particular, outlining structured physical therapy regimens used in conjunction with gabapentinoids. Specific exercises, intensities, durations, and progressions should be reported. This will allow an analysis of which integrated programs demonstrate the greatest benefit. By combining pharmacological options such as gabapentinoids with structured physical therapy and activity modification, the need for invasive procedures can be delayed.

### 4.1 Limitations

Several limitations were identified in this study. Firstly, the absence of previous meta-analyses made it difficult to compare our results and determine their reliability. Additionally, the limited number of publications on the treatment of LSS with gabapentinoids posed challenges. Other limitations included differences in time records and follow-up periods among the trials, as well as variations in inclusion criteria and the use of different measurement scales, which made it challenging to conduct a valid comparison across studies. Furthermore, the control group in some studies used different drugs, and one study had a different design from the randomized controlled trials. Additionally, one study focused on cervical spinal stenosis, although the drug is expected to act similarly at any level of the spine. The small sample size precluded the use of sensitivity analysis, and publication bias through funnel plots was inconsistent.

## 5 Conclusion

The meta-analysis findings suggest that, overall, pregabalin and gabapentin did not demonstrate a greater reduction in pain compared to NSAIDs, limaprost, or diphenhydramine in the short term. However, these drugs appeared to be more effective in the medium term in reducing pain scores obtained through the VAS/NRS. Moreover, there was no evidence to suggest that gabapentinoids were more effective in reducing disability in patients, as measured through the ODI, compared to the drugs in the control group. The study did indicate that pregabalin and gabapentin had a worse safety profile, with a higher incidence of adverse events compared to other drugs. The limited results obtained in this meta-analysis underscore the need for additional clinical trials and comparative studieswith unified evaluation criteria and standardized reporting of the temporal evolution of symptoms”.

## Data Availability

The original contributions presented in the study are included in the article/Supplementary material, further inquiries can be directed to the corresponding author.
